# Identification of hypertrophy-modulating Cullin-RING ubiquitin ligases in primary cardiomyocytes

**DOI:** 10.3389/fphys.2023.1134339

**Published:** 2023-03-08

**Authors:** Maximillian Fischer, Moritz Jakab, Marc N. Hirt, Tessa R. Werner, Stefan Engelhardt, Antonio Sarikas

**Affiliations:** ^1^ Institute of Pharmacology and Toxicology, Technische Universität München, Munich, Germany; ^2^ DZHK (German Center for Cardiovascular Research), Partner Site Munich Heart Alliance, Munich, Germany; ^3^ Institute of Pharmacology, University Medical Center Hamburg-Eppendorf, Hamburg, Germany; ^4^ DZHK (German Center for Cardiovascular Research), Partner Site Hamburg/Kiel/Lübeck, Hamburg, Germany; ^5^ Institute of Pharmacology and Toxicology, Paracelsus Medical University, Salzburg, Austria

**Keywords:** cardiomyocyte, hypertrophy, phenotypic screen, Cullin-RING E3 ligase (CRL), F-box protein, Fbxo25

## Abstract

Cullin-RING ubiquitin ligases (CRL) regulate numerous biological processes in the heart and have been implicated in regulating cardiac hypertrophy. This study aimed to identify novel hypertrophy-modulating CRLs in cardiomyocytes (CM). A functional genomic approach using siRNA-mediated depletion and automated microscopy was employed to screen for cell size-modulating CRLs in neonatal rat CM. Screening hits were confirmed by ^3^H-isoleucine incorporation. Of 43 targets screened, siRNA-mediated depletion of Fbxo6, Fbxo45, and Fbxl14 resulted in decreased cell size, whereas depletion of Fbxo9, Fbxo25, Fbxo30, Fbxo32, Fbxo33, Cullin1, Roc1, Ddb1, Fbxw4, and Fbxw5 led to a markedly increased cell size under basal conditions. In CM stimulated with phenylephrine (PE), depletion of Fbxo6, Fbxo25, Fbxo33, Fbxo45, and Fbxw4 further augmented PE-induced hypertrophy. As a proof-of-concept, the CRL^Fbox25^ was analysed by transverse aortic constriction (TAC) resulting in a 4.5-fold increase in Fbxo25 protein concentrations compared to control animals. In cell culture, siRNA-mediated depletion of Fbxo25 resulted in a ∼ 37% increase in CM cell size and ∼41% increase in ^3^H-isoleucine incorporation. Depleting Fbxo25 resulted in upregulation of Anp and Bnp. In summary, we identified 13 novel CRLs as positive or negative regulators of CM hypertrophy. Of these, CRL^Fbox25^ was further characterized, as a potential modulator of cardiac hypertrophy.

## 1 Introduction

Cardiac hypertrophy is a frequent hallmark of cardiovascular diseases and represents an independent risk factor for cardiac morbidity and mortality [reviewed in (Nakamura and Sadoshima, 2018)]. While hypertrophy of cardiomyocytes (CM) is initially a compensatory response to counteract increased biomechanical loading of the ventricle (e.g., by pressure or volume overload), prolonged hypertrophy may become detrimental and result in maladaptation and heart failure when pro-hypertrophic stimulation persists [reviewed in (Nakamura and Sadoshima, 2018)].

The pathophysiology of cardiac hypertrophy is complex and involves the activation of multiple signalling pathways that control CM growth, resulting in increased CM size, reactivation of fetal gene programme and altered signal transduction pathways [reviewed in (Heineke and Molkentin, 2006; Van Berlo et al., 2013)]. Emerging evidence suggests a vital role of the ubiquitin-proteasome system (UPS) in the pathogenesis of cardiovascular diseases, particularly cardiac hypertrophy (Zolk et al., 2006; Mearini et al., 2008). Central to the UPS is the recognition of a substrate by an E3 ubiquitin ligase, a step pivotal for the ubiquitin-mediated degradation of substrate proteins by the 26S proteasome (Nath and Shadan, 2009).

Cullin-RING complexes (CRLs) constitute the largest group of E3 ligases, which are characterized by two signature components: a cullin (CUL) scaffold protein and the RING (for Really Interesting New Gene) finger protein ROC1. In the prototypic SCF (Skp1•CUL1•F-box protein•ROC1) complex, the CUL1 N-terminus binds to the Skp1•F-box protein substrate-targeting module, whereas the C-terminally located cullin domain anchors ROC1, which recruits E2 conjugating enzyme to catalyze the transfer of Ub to the substrate protein [reviewed in (Sarikas et al., 2011)].

SCF contains a substrate recognition subunit known as the F-box protein, characterized by a 40-amino-acid F-box domain (Skaar et al., 2013). F-box proteins are interchangeable components of the SCF type E3 ligases and dictate the ubiquitin ligase’s substrate specificity. Of the 69 F-box proteins encoded by the human genome, only a fraction has been studied in the heart.

Several E3 ubiquitin ligases have been identified as critical regulators of cardiac hypertrophy. E3 ligases have been shown to be muscle enriched or muscle-specific, such as muscle-specific RING finger protein (MuRF)-family, Atrogin-1, carboxyl terminus of HSP70-interacting protein (CHIP) (Zhang et al., 2005) or the SCF containing the F-box and leucine-rich repeat protein 22 (Fbxl22) (Spaich et al., 2012; Hughes et al., 2020). The RING-type E3 ubiquitin ligase MuRF1, also known as TRIM63, was first identified as a mediator of muscle atrophy (Bodine et al., 2001) and shown to target sarcomeric structural proteins, e.g., myosin heavy chain protein (Clarke et al., 2007; Fielitz et al., 2007), troponin I (Kedar et al., 2004) or myosin-binding protein C (Mearini et al., 2010) for proteasomal degradation. In addition, MuRF1 inhibits transcription factors critical for cardiac hypertrophy, e.g., serum response factor (Willis et al., 2007) and c-Jun (Wadosky et al., 2014). Another muscle-specific ubiquitin ligase is the SCF containing the F-box protein Fbxo32 (also known as Atrogin-1 or MAFbx). Atrogin-1 forms an SCF complex to ubiquitinate different transcription factors, such as nuclear factor of activated T cells (Li et al., 2004) and Forkhead transcription factors downstream of AKT (Li et al., 2007). Transgenic mice overexpressing Atrogin-1 in the heart demonstrated a blunted cardiac hypertrophy in a pathophysiological model of transverse aortic constriction (TAC) by reduction of calcineurin levels (Li et al., 2004). In contrast, genetic ablation of Atrogin-1 in the heart resulted in exaggerated cardiac hypertrophy (Li et al., 2007). These studies not only validated the relevance of ubiquitin ligases for cardiac hypertrophy but also paved the way for the UPS as a pharmacological target for preventing or treating cardiac diseases (Drews and Taegtmeyer, 2014; Drews, 2016; Bowen et al., 2017).

In the present study, we established a siRNA-mediated screening assay in neonatal rat cardiomyocytes (NRCM) and used this approach to identify cardiac hypertrophy-modulating CRL ligases.

## 2 Material and methods

### 2.1 Antibodies and reagents

For immunoblotting and histology, the following primary antibodies were used: anti-α-Actinin (sarcomeric) (A7811, Sigma Aldrich), anti-FBXO25 (gift from AG Bassermann (TUM MRI), anti-HSP90 α/β (Sc-13119, Santa Cruz). Secondary antibodies used: Goat anti-mouse IgG (H + L) Alexa 488 (A11029, Invitrogen), Goat anti-rabbit IgG (H + L) Alexa 488 (A11034, Invitrogen), anti-mouse HRP (7074S, Cell Signaling), anti-rabbit HRP (7076S, Cell Signaling). Penicillin, streptomycin, fetal bovine serum (FBS) and other reagents were obtained from Invitrogen Life Technologies (Carlsbad, CA) or Sigma-Aldrich (Louis, MO).

### 2.2 Isolation neonatal rat cardiomyocytes

Adult rats were anaesthetized with isoflurane (4%), followed by intraperitoneal injection of 80 mg/kg xylazine and 12 mg/kg ketamine. The depth of anaesthesia was monitored by toe pinch. Neonatal hearts were isolated from 1 to 2-day-old Sprague Dawley rats after decapitation. All procedures were in accordance with the Guide for the Care and Use of Laboratory Animals published by the United States National Institutes of Health and approved by local authorities.

### 2.3 Transfection of neonatal rat cardiomyocytes

The transfection of neonatal rat cardiomyocytes was performed as described previously (Jentzsch et al., 2012). In brief: ON-TARGETplus rat siRNA (Supplementary Table S4) was diluted to 20 μM and then stored at −20°C. The concentration was confirmed using Nanodrop (absorbance at 260 nm, RNA-40). Transfection of NRCM with siRNA was performed in 96 well plate dishes according to the following protocol: prewarmed Opti-MEM I and 5% FBS NRCM medium without antibiotics PenStrep were prepared freshly before transfection. Transfection samples were prepared by mixing and diluting the volume of siRNAs with Opti-MEM I. Next, the transfection reagent Dharmafect 1 was prepared by mixing and incubation for 20 min at room temperature. The NRCMs were incubated at 37°C and 1% CO2 for 4 to 6 h. After incubation, the medium was changed to 200 μL 0.1% FBS NRCM medium with antibiotics, and cells were cultured (37°C and 1% CO2) for 48 h.

### 2.4 Genome expression array

Analysis of the transcribed mouse genome and corresponding expression levels were carried out on cardiac RNA samples (500 ng input) using the Affymetrix GeneChip^®^ Mouse Expression Set 430, as described previously (Busch et al., 2002; Han et al., 2002; Ueda et al., 2002). Mean expression levels are displayed in log_2_ scale according to manufacturer protocol (Supplementary Figure S1). Expression levels starting at 6.0 provide relevant protein synthesis and above at 6.5 argues for strong protein expression, according to the manufacturer. Data are available by GEO accession number: GSE224276.

### 2.5 Immunostaining

Cells were fixed with 4% paraformaldehyde (5 min) and permeabilized with 0.2% Triton-X (5 min) at room temperature. We performed cardiomyocyte-specific immunostaining using an antibody that recognizes sarcomeric α-actinin (1:1,000; clone EA-53, Sigma-Aldrich, Taufkirchen, Germany) and, as a secondary antibody, Alexa 488-coupled α-mouse antibody (1:200; Invitrogen, Karlsruhe, Germany); nuclei were stained by 4′,6-diamidin-2-phenylindol (DAPI) (1 μg/μL final concentration; Sigma-Aldrich, Taufkirchen, Germany). All antibody incubation steps were performed at 37°C and intermitted by three washing steps with PBS. Finally, cells were covered with 50% glycerol and stored at 4°C until microscopical analysis.

### 2.6 Automated cell size and nuclei count determination

Cell size determination was established as a fully automated microscopic procedure from scanning 96 well plates to analyzing the acquired data sets regarding cell size and number as described previously (Jentzsch et al., 2012). The following setup was used for image acquisition with a ×10 objective: AxioObserver.Z1 (Zeiss, Jena, Germany), motorized scanning stage (130 × 85; Märzhäuser, Wetzlar, Germany), Lumen200 fluorescence illumination system (Prior, Cambridge, United Kingdom), and Retiga-4000DC CCD camera (QImaging, Surrey, Canada). We automated image acquisition and analysis *via* macro functions (termed journal mode) of the MetaMorph Basic imaging software package (Molecular Devices, Downingtown, United States). One master journal comprised three sub-journals for focusing, acquisition, and data processing.

### 2.7 Isoleucine incorporation assay

48 h after transfection, ^3^H-isoleucine (Hartmann Analytic, Braunschweig, Germany) was added to the NRCM culture medium described above (final concentration: 1 μCi/mL). Cells were stimulated with 50 μM phenylephrine (PE) as a positive control for 48 h. Cells were then washed with PBS and incubated with 5% trichloroacetic acid for 1 h on ice. Cells were subsequently lysed in 0.5 M NaOH for 30 min at 37°C, and lysates were mixed with 10 mL scintillation fluid (Roth, Karlsruhe, Germany) for quantitation of ^3^H.

### 2.8 Quantitative real-time PCR

Quantitative real-time PCR Total RNA was prepared using PeqGOLD RNApure (Peqlab) according to the manufacturer’s protocol. 500 ng RNA was reverse transcribed using the Protoscript II cDNA Synthesis Kit (NEB, procedure according to manufacturer’s protocol). Quantitative PCR analysis was conducted using the Fast Start SybrGreen MasterMix according to the standard protocol (Roche). Please find Primer sequences for real-time PCR (gene symbols and species followed by sequences of forward and reverse primers) in Supplementary Table S3.

### 2.9 MTT assay

96 h after transfection, 8 mg MTT was dissolved in 500 µL of PBS. 400 μL of the MTT solution was added into 12 mL of 0% FBS NRCM medium, mixed and prewarmed in the water bath at 37°C. NRCM were incubated with 400 µL of the 0% FBS NRCM medium with MTT and incubated at 37°C and 1% CO2 for 1 h. After incubation, the NRCMs were washed once with 1xPBS, and 200 µL of acidified isopropanol (10 mL isopropanol with 90 µL concentrated HCL) was added. NRCMs were lysed by pipetting. The content of each well was transferred into a transparent 96well plate, and the absorbance was measured in a plate reader at 570 nm and the background absorbance at 650 nm subtracted.

### 2.10 Immunoprecipitation and western blot analysis

Immunoprecipitations and Western blot analyses were performed as described previously (Hartmann et al., 2014; Scheufele et al., 2014). In brief: protein extraction was performed in lysis buffer containing protease and phosphatase inhibitors (Roche) and immunoblots performed using standard procedures. Protein lysates were electrophoresed on 10% or 12% SDS-PAGE gels, transferred onto a PVDF membrane and blocked with bovine serum albumin for 2 h at room temperature.

### 2.11 Transverse aortic constriction model

Transverse aortic constriction (TAC) is an established model of pressure overload in the heart and mimics cardiovascular diseases in humans, such as cardiac hypertrophy. TAC surgery was performed on 8-week-old male C57BL/6 N mice (Charles River Laboratories), as described previously (Rockman et al., 1991). In brief: mice were injected intraperitoneally with buprenorphine (0.08 mg/kg) and metamizole (200 mg/kg) for analgesia. Mice were anesthetized in a narcosis chamber with 4% isoflurane mixed with 0.5 L/min of 100% O2. During the surgical procedure, isoflurane anaesthesia was maintained at 1.5%–2% isoflurane with 0.5 L/min O2. Partial thoracotomy was performed, and a 6.0 silk suture was ligated between the innominate and left carotid arteries around a 27.5-gauge needle placed parallel to the transverse aorta. In sham surgery, the chest was opened, but no ligation of the aorta was performed (Ramanujam et al., 2021). All animal studies were performed in accordance with the relevant guidelines and regulations of the responsible authorities. Mice were sacrificed 2 and 6 weeks after surgery, respectively.

### 2.12 Statistical analysis

Data are presented as mean ± SEM unless stated otherwise. Statistical analysis was performed with Prism (Version 8, GraphPad Software, Inc., San Diego, California). A 2-tailed Student t-test assessed differences between the two means for Gaussian distributed values. Differences among multiple means were assessed by 1-way ANOVA followed by Bonferroni *post hoc*, assuming Gaussian distribution of the measured parameters. *N* = independent experiments/cell preparations, *n* = replicates. Differences were considered significant when *p* < 0.001 (***), <0.01 (**) or <0.05 (*).

## 3 Results

### 3.1 siRNA-based screening to identify hypertrophy-modulating ubiquitin ligases in primary neonatal rat cardiomyocytes

**FIGURE 1:**
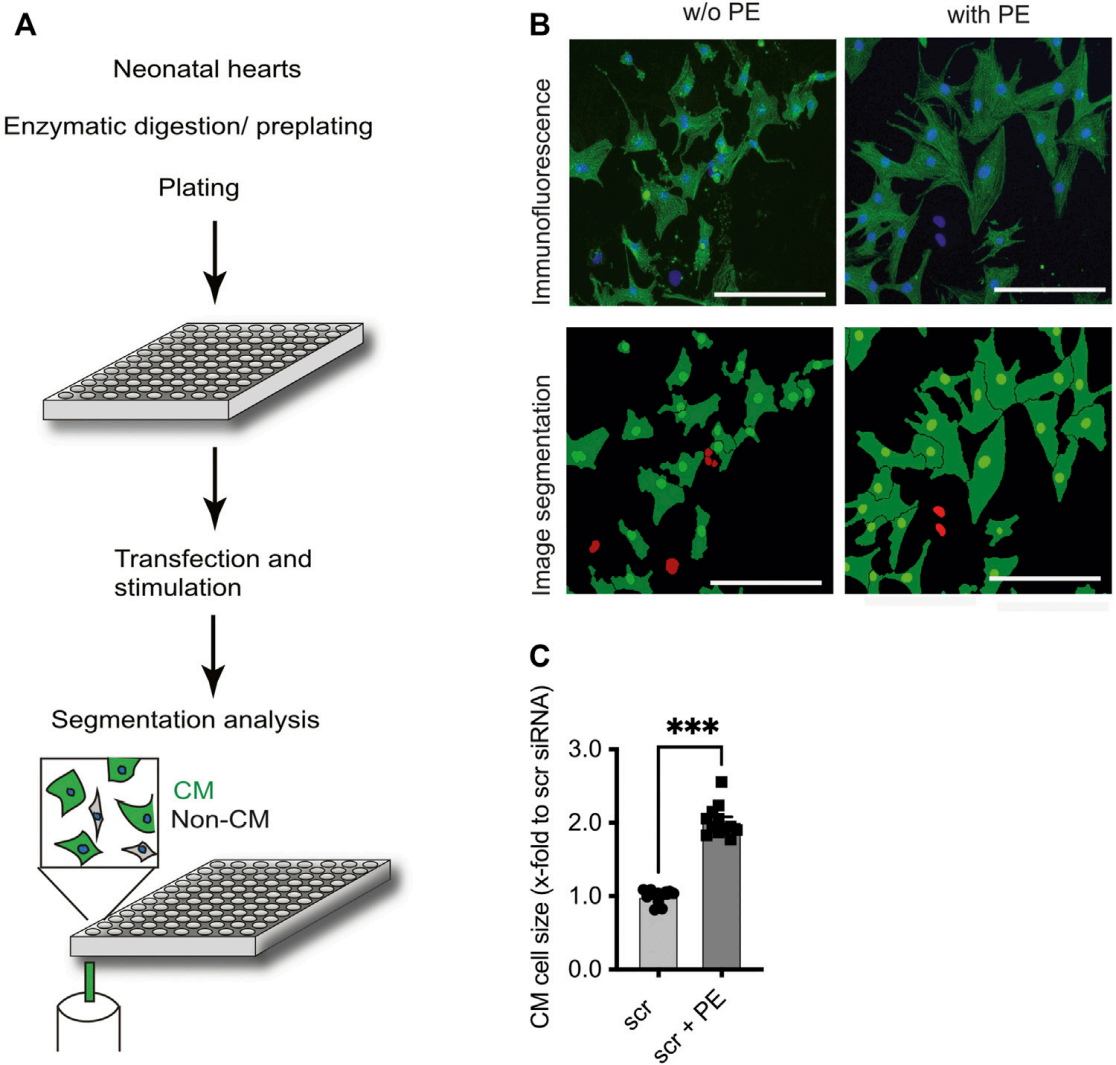
Identification of F-box protein Fbxo25 as a novel regulator of cardiomyocyte hypertrophy. **(A)** Schematic workflow: Cells were isolated from neonatal rat hearts, pre-plated to enrich for cardiomyocytes, and seeded onto 96 well plates. After transfection with 43 different siRNAs, cell identity was determined by immunostaining against α-actinin and DAPI. Cell size was quantitated by automated microscopy and image segmentation. **(B)** Representative immunostaining and image segmentation of neonatal cardiomyocytes in cell culture; cardiomyocytes (α-actinin) were stained for the marker protein. Nuclei stained with DAPI in blue in immunofluorescence picture. Non-CM are labelled red in image segmentations. Scale bar represents 250 μm. **(C)** Histograms of the effect after siRNA scramble transfection on cardiomyocyte size under basal conditions or stimulation with phenylephrine (PE) 50 µM. Dots depict cumulative data from three independent cell preparations. Each independent experiment with > 50,000 cells per group. Data are normalized to scrambled siRNA control. Independent experiments: *N* = 3, *n* = 12, unpaired Students t-test, ***p < 0.001.

An adapted protocol for cell size determination by automated microscopy, first described by Jentzsch and colleagues (Jentzsch et al., 2012) was employed to screen for hypertrophy-modulating CRLs in primary neonatal rat cardiomyocytes (NRCM) (Figures 1A). A library of siRNA was chosen to deplete single CRLs in NRCM selectively. Only siRNA directed against CRLs or CRL components with high mRNA expression levels in CMs based on microarray profiling data (Supplementary Figure S1) were included in the siRNA library (See Supplementary Table S1). Analysis of the Netaffx microarray data resulted in three groups of targets according to their expression level. For values >6.0 relevant gene expression (and protein synthesis) is likely, and >6.5 argues for strong protein synthesis, according to manufacturer. Group 1 included the highest expressed targets with a mean value greater than 6.5 in multiple measurements. The second group with values in multiple measurements and mean values between 6 and 6.49 on the log_2_ scale. The third group includes components of the Cullin-RING E3 ligases (e.g., backbone proteins and adaptors) for control reactions by knocking down essential subunits. Cell size measurements were conducted in 96 well format with scramble and siRNA-treated cells. The CRL containing Fbxo32 (also known as Atrogin-1) served as internal control for internal validation (Supplementary Figure S2A). PE-stimulated cardiomyocytes showed consistent hypertrophy (Supplementary Figure S2B). Realtime-PCR revealed a knockdown of ∼80–90%, *p* < 0.05 in student’s *t*-Test for all targets investigated (Supplementary Figure S3). MTT and DAPI nuclei count assays monitored the toxicity of siRNA transfection during the screening. No significant cell toxicity was observed upon transfection of the siRNA library (Supplementary Figures S4, S5). Our results indicate that NRCMs can be transfected with a siRNA library directed against CRLs in NRMCs and monitored for their effect on cardiomyocyte size.

### 3.2 Identification of Cullin-RING ubiquitin ligases regulating cardiomyocyte hypertrophy under basal and pro-hypertrophic conditions

**FIGURE 2:**
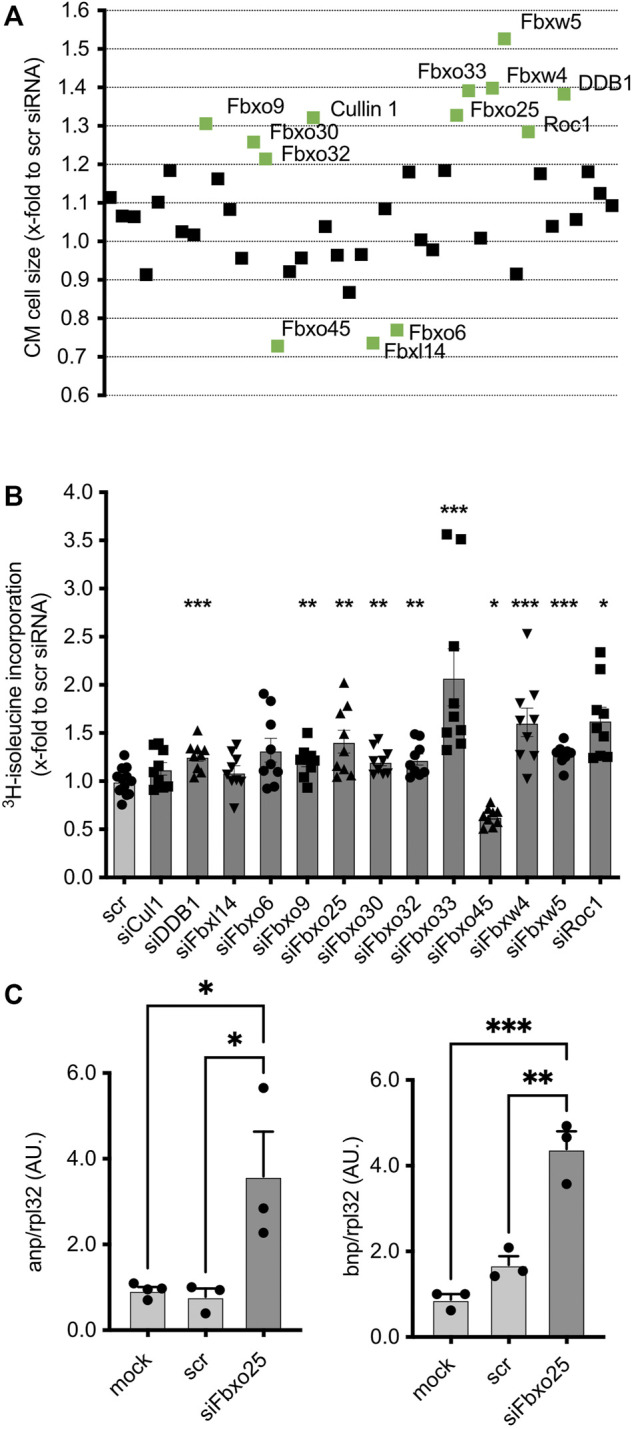
Effect of transfected siRNAs on CM at basal conditions. **(A)** Effect of transfected siRNAs on CM size upon basal condition without PE. Data are normalized to scramble control (scr). Screening targets are depicted on the *x*-axis. Three independent experiments with > 50,000 cells per group. Statistical significance between the screening targets was analyzed by 1-way ANOVA and Bonferroni *post hoc test*. The labelled screening hits are highly significant (labelled in green). **(B)** Validation of cell size changing siRNA-mediated knockdown effect on cardiomyocytes by 3H- isoleucine incorporation without PE. Data were normalized to scramble siRNA control. Dots depict cumulative data from three independent cell preparations. Independent experiments: *N* = 3, *n* = 10–11. Histogram shows data under basal conditions without phenylephrine (PE). Data are mean ± SEM. One-way ANOVA with Bonferroni *post hoc* test, * *p* < 0.05, ** *p* < 0.01, *** *p* < 0.001. **(C)** mRNA concentrations of hypertrophy markers atrial natriuretic peptide (ANP, left panel) and brain natriuretic peptide (BNP, right panel) 96 h after transfection of NRCMs. All data shown were normalized to Rpl32. *N* = 3, 4 with > 50,000 cells each. *p < 0.05, ***p < 0.001, 1-way ANOVA with Bonferroni *post hoc test.*

In line with previous reports (Jentzsch et al., 2012), incubation of NRCM with the α_1_-adrenergic receptor agonist phenylephrine (PE, 50 µM) for 48 h resulted in a ∼2-fold increase (2.02 ± 0.06, *p* < 0.001) of CM cell size in comparison to mock (PBS) treated CM (Figures 1B, C). Figure 2A displays screening results as a fold change of the mean cell size upon transfection of siRNAs directed against CRLs compared to the mean of scramble control siRNA-transfected cells included on each plate at basal conditions (see Supplementary Table S2 for a tabular presentation).

Of all 43 siRNAs screened, ∼32% (14 siRNAs) induced significant (*p* < 0.05) change in cell size in comparison to the control group (Figure 2A). Of these, Fbxo32 (1.21 ± 0.02, *p* < 0.05), Fbxo9 (1.31 ± 0.05, *p* < 0.001), Cullin1 (1.32 ± 0.05, *p* < 0.001), Fbxo25 (1.37 ± 0.03, *p* < 0.001), Fbxo30 (1.26 ± 0.04, *p* < 0.01), Roc1 (1.29 ± 0.03, *p* < 0.001), Fbxo33 (1.39 ± 0.06, *p* < 0.001), Fbxw4 (1.39 ± 0.02, *p* < 0.001), Fbxw5 (1.52 ± 0.05, *p* < 0.001) and Ddb1 (1.38 ± 0.05, *p* < 0.001), showed the most pronounced effect. Of note, depletion of Fbxo45 (0.73 ± 0.03, *p* < 0.01), Fbxl14 (0.76 ± 0.02, *p* < 0.01) and Fbxo6 (0.77 ± 0.03, *p* < 0.05) resulted in a marked reduction of mean cell size. All screening hits were analyzed by ^3^H-isoleucine incorporation, an established indicator for nascent protein synthesis accompanying hypertrophy (Figure 2B). The majority of the screened targets, including Fbxo32 (1.23 ± 0.06, *p* < 0.01), Fbxo9 (1.20 ± 0.05, *p* < 0.01), Cullin1 (1.13 ± 0.07, n.s.), Fbxo25 (1.41 ± 0.12, *p* < 0.01), Fbxo30 (1.21 ± 0.05, *p* < 0.01), Fbxo33 (2.08 ± 0.29, *p* < 0.001), Fbxw4 (1.61 ± 0.15, *p* < 0.001), Fbxw5 (1.28 ± 0.03, *p* < 0.001) and Ddb1 (1.26 ± 0.05, n.s.), showed enhanced ^3^H-isoleucine incorporation. Of note, only after depletion of Fbxo45 (0.63 ± 0.03, *p* < 0.05) bot not of Fbxl14 (1.01 ± 0.07, n.s.) and Fbxo6 (1.32 ± 0.12, n.s.), this assay showed a hypotrophy correlate, which might be due to other unknown intracellular metabolic mechanisms.

**FIGURE 3:**
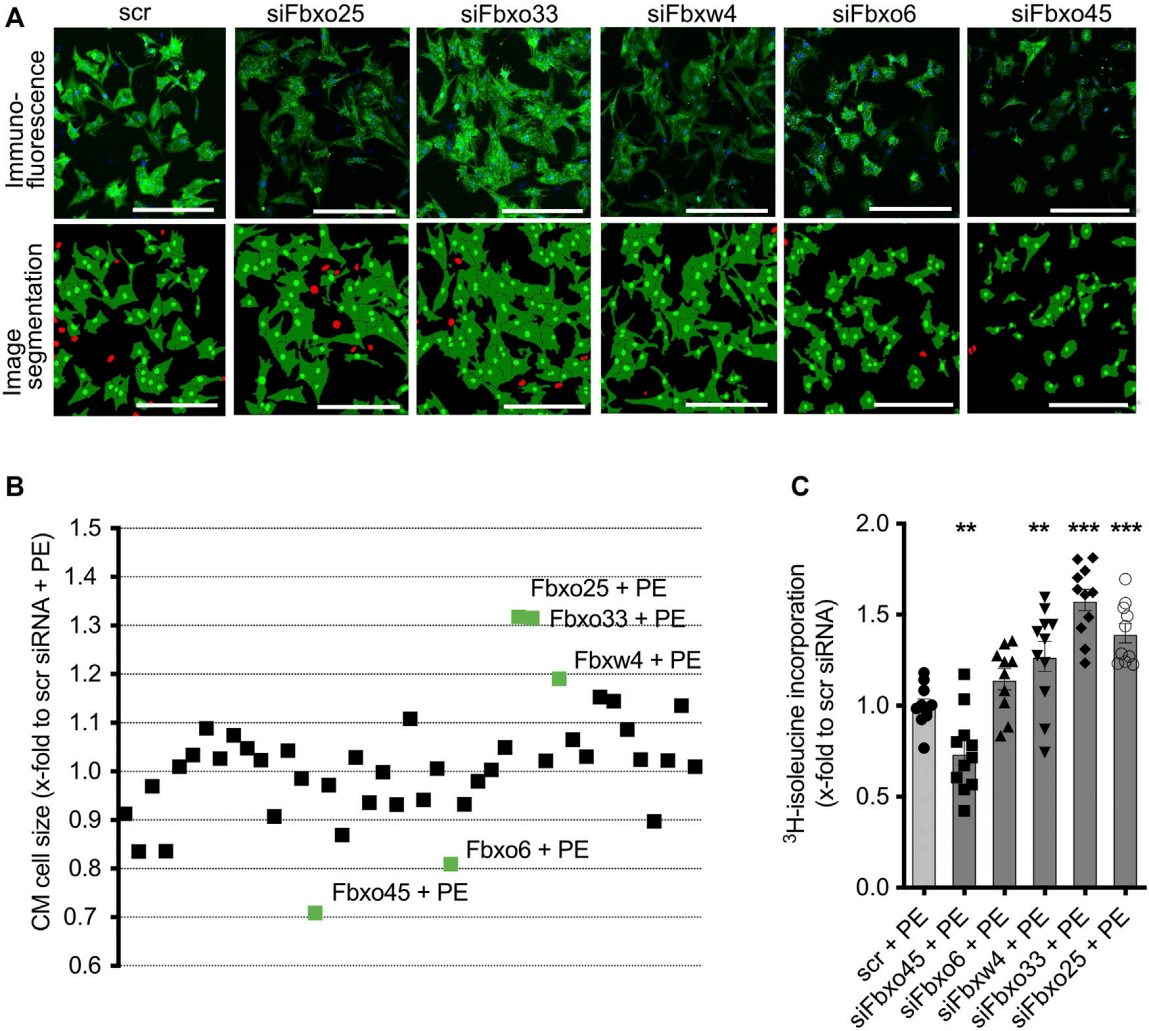
Effect of transfected siRNAs on CM at PE-induced CM hypertrophy. **(A)** Representative immunostainings and image segmentations of neonatal rat cardiomyocytes (CM) upon transfection with scramble control or siRNAs against Fbxo25, Fbxo33, Fbxw4, Fbxo6, and Fbxo45. CM were identified by staining for α-actinin (green) and nuclei are labelled with DAPI (blue) in immunofluorescence pictures. Non-CM are labelled red in image segmentations. Scale bar = 500 μm. **(B)** Effect of transfected siRNAs on CM size upon PE-induced CM hypertrophy. Data are normalized to scramble control with PE. Three independent experiments with > 50,000 cells per group. Statistical significance between the screening targets was analyzed by 1-way ANOVA and Bonferroni *post hoc test.* The labelled screening hits are highly significant (labelled in green). **(C)** Validation of cell size change after siRNA transfection on CM by ^3^H- isoleucine incorporation with PE-induced hypertrophy. Data are normalized to scramble control with PE (scr + PE). Dots depict cumulative data from three independent cell preparations. Three independent experiments: *N* = 3, *n* = 10–11. Statistical significance between the screening targets was analyzed by 1-way ANOVA and Bonferroni *post hoc test.*

Next, we evaluated the effect of siRNA-mediated depletion of CRLs in hypertrophic NRCM (Figures 3A, B; see Supplementary Table S2 for a tabular presentation). Depletion of Fbxo25 (1.32 ± 0.03, *p* < 0.001), Fbxo33 (1.31 ± 0.04, *p* < 0.001) and Fbxw4 (1.19 ± 0.03, *p* < 0.001) resulted in significant additional increase of mean cell size of PE-treated NRCMs. On the contrary, knockdown of Fbxo45 (0.71 ± 0.02, *p* < 0.001) and Fbxo6 (0.81 ± 0.02, *p* < 0.001) resulted in approx. 20%–30% decrease in mean cell size of hypertrophic NRCMs. All significant results und PE stimulation were analysed by ^3^H-isoleucine incorporation (Figure 3C). Of these, Fbxo25 (1.40 ± 0.06, *p* < 0.001), Fbxo33 (1.58 ± 0.06, *p* < 0.001) and Fbxw4 (1.27 ± 0.08, *p* < 0.001) resulted in significant ^3^H-isoleucine incorporation. In line with the previous mediated hypotrophy, Fbxo45 (0.74 ± 0.07, *p* < 0.01) and Fbxo6 (1.15 ± 0.06, ns) were analyzed for ^3^H-isoleucine incorporation after depletion. Supplementary Figure S2C displays the screening results at basal and PE stimulation normalized to untreated scramble control.

### 3.3 Loss of Fbxo25 results in cardiomyocyte hypertrophy

As a proof-of-concept for the validity of the screening assay, we further studied the role of SCF Fbxo25. Previous publication showed, that Fbxo25 promotes the degradation of growth factors such as ELK-1 (Teixeira et al., 2013), and is destructing cardiac-specific transcriptions factors (Jang et al., 2011; Jeong et al., 2015). Depletion of F-box protein Fbxo25, as a CRL target with significant cell size increase upon depletion both at basal and under PE stimulation (Figures 2A, 3B). The pro-hypertrophic effect by ∼32% upon Fbxo25 depletion was confirmed by ^3^H-isoleucine incorporation (Figures 2B, 3C).

### 3.4 Increased FBXO25 protein abundance in pathological hypertrophic heart tissue upon transverse aortic constriction and increased ANP/BNP mRNA upon Fbxo25 depletion

**FIGURE 4:**
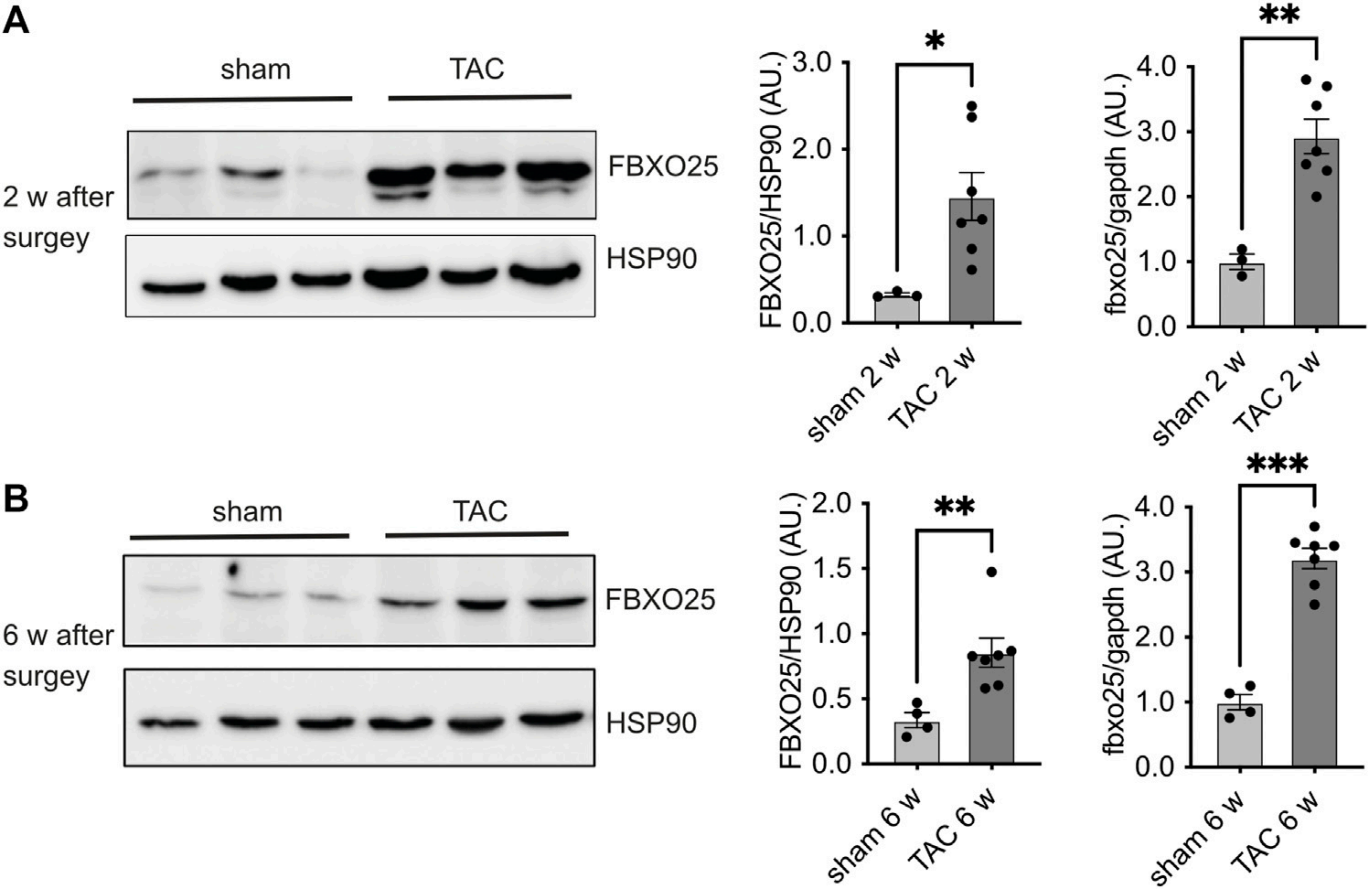
Fbxo25 accumulates in murine hearts upon transverse aortic constriction (TAC). Immunoblot of FBXO25 protein level, mRNA levels and quantification thereof in murine hearts lysates after two [upper panel **(A)**] and 6 weeks [lower panel **(B)**] upon TAC. HSP90 served as a protein loading control. Quantification of mRNA levels after two and 6 weeks (right panel) are normalized to control and gapdh served as housekeeping gene. Sham-operated littermates served as controls, *N* = 3–7. Histograms depict quantitative data (right panel). Statistical significance was determined using unpaired student’s t-test: *p < 0.05; **p < 0.01.

To investigate the abundance of Fbxo25 *in vivo*, we analyzed the FBXO25 protein in heart samples of mice undergoing transverse aortic constriction (TAC), a model resulting in pressure overload and hypertrophy. We observed upregulation of FBXO25 protein abundance 2 weeks (∼4.5-fold, *p* < 0.05) and 6 weeks (∼2.5-fold, *p* < 0.01) after TAC when compared to sham controls (Figures 4A, B). Next, we evaluated the mRNA levels of Fbxo25 in TAC heart (Figure 4, right panel). Fbxo25 mRNA levels increased at 2 weeks (*p* < 0.01) and maintained ∼3-fold higher after 6 weeks (*p* < 0.001).

Furthermore, the transfection of siRNA-mediated depletion of Fbxo25 led to a marked increase of atrial natriuretic factor (Anp) and brain natriuretic factor (Bnp) transcripts, a hallmark of the hypertrophy-associated gene expression program in NRMCs (Figure 2C). *ANP* mRNA levels were increased 4.6-fold, and *BNP* mRNA levels increased 2.6-fold when compared to scramble-siRNA controls (*N* = 3-4, *p* < 0.05). Taken together, these data give novel insight that into CRLs subunits playing a yet unrecognized role in CM.

## 4 Discussion

Our screening showed a statistically significant increase in CM cell size after knockdown of Fbxo9, Fbxo25, Fbxo30, Fbxo32, Fbxo33, Cullin1, Roc1, Fbxw4, Fbxw5, and Ddb1, whereas the knockdown of Fbxo6, Fbxo45, and Fbxl14 resulted in a decrease in CM cell size. After phenylephrine induced hypertrophy in NRCMs, knockdown of Fbxo6, Fbxo25, Fbxo33, Fbxo45, and Fbxw4 resulted in significant statistically changes in cell size.

Using this unbiased approach, we identified several members of CRLs, besides Atrogin-1 (Fbxo32), that has been studied extensively (Bodine et al., 2001; Lecker et al., 2004), of which the majority had not been implicated in CM hypertrophy before. Atrogin-1 was shown to inhibit cardiac hypertrophy *via* targeting of calcineurin/nucleus factor of activated T (NFAT) and phosphatidylinositol-3 (PI-3)/Akt/Foxo signalling pathways (Li et al., 2004; Li et al., 2007; Skurk et al., 2005). In contrast, the deficiency of Atrogin-1 leads to augmented cardiac hypertrophy *in vivo* (Li et al., 2007). Recently, studies also identified the Atrogin-1 gene as a new player in familial dilatative cardiomyopathy (Al-Yacoub et al., 2016) and described its mechanistic interaction with the endoplasmatic reticulum-stress apoptosis protein called CHOP (C/EBP homologous protein) (Al-Yacoub et al., 2021).

Throughout our siRNA-based screening in CM, we used the characterized Atrogin-1 as a valid prototype and internal control to evaluate our approach. Similarly, siRNA-mediated knockdown of Atrogin-1 mRNA resulted in a hypertrophic response in CM shown by immunofluorescence microscopy and ^3^H-isoleucine incorporation. In this study, we used a relative mRNA expression assessed from microarray data. It should be pointed out that relatively low expression levels of a given protein do not necessarily mean it is functionally less important. A single expression analyses could also potentially miss other relevant CRLs in the heart.

Fbxo32 (Atrogin-1), a key regulator of cardiac hypertrophy (Li et al., 2004; Li et al., 2007), was identified by our screening approach and showed a 1.21-fold increase in mean cell size upon siRNA-mediated depletion at basal conditions (Supplementary Figure S2). Besides Atrogin-1, we observed other CRLs containing the F-box motif that showed an even more substantial hypertrophic effect after siRNA-mediated depletion in CMs. Of these, the F-box protein 25, was identified as a potential regulator in our screening. Teixeira et al. showed that Fbxo25 promotes the degradation of ELK-1, which is known as the regulator of c-fos (Teixeira et al., 2013). Remarkably, Fbxo25 acts as an E3 ligase for destructing cardiac-specific transcriptions factors (e.g., Nkx2-5, Isl1, Hand1, and Mef2C) with higher expression levels in fetal than in adult heart cells (Jang et al., 2011). Recently, Baumann et al. showed that Hax-1 protein, a well-known player in the heart, is targeted by CRL containing Fbxo25 (Baumann et al., 2014). Hax-1, primarily described as a multifunctional protein (Han et al., 2006; Shaw and Kirshenbaum, 2006; Vafiadaki et al., 2009; Lam et al., 2013; Mattiazzi and Kranias, 2014), was identified as a regulator of myocardial contractility and calcium cycling (Zhao et al., 2009). As a proof-of-principle, we evaluated the Fbxo25 in different models: neonatal cardiomyocytes, and *in vivo* model of pathologic cardiac hypertrophy upon transverse aortic constriction.

In the TAC model, we observed a significant increase in Fbxo25 protein and mRNA levels after 2 and 4 weeks of pathologically increased cardiac afterload. Our data show that siRNA-mediated knockdown of Fbxo25 results in CM hypertrophy which is associated with increased BNP and ANP mRNA levels. Therefore, Fbxo25 might display a counteractive regulation in heart hypertrophy. Future studies are warranted to investigate, if the loss of Fbxo25 in CM results in augmented hypertrophy and Fbxo25 overexpression diminishes hypertrophy by acting as a potential counterregulatory protein. However, if the presence and deficiency are equally important for its cellular function remains to be elucidated in future studies. These results suggest a potential role of Fbxo25 in heart hypertrophy and underline the putative role of the other CRL subunits identified in our screening. Regarding the limitations of the study, we combined experiments using PE, an inductor of pathological *in vitro* hypertrophy, and a TAC model for the same target. Therefore, our study suggests yet unknown UPS subunits to play a potential role in cardiac hypertrophy, however due to our experimental design, our results can’t assign our screening hits, e.g., Fbxo25 to either physiological or pathophysiological cardiac hypertrophy. Our screening showed a significant effect on CM cell size after the knockdown of several other targets, including Fbxo9, Fbxo30, Fbxo33, Fbxw4, Fbxw5, and Ddb1 as well for the scaffold proteins of the CRL (Roc1 and Cul1). The Cul1 protein acts as a scaffold, anchoring the RING finger protein and the adaptor for the attachment of the variable F-box proteins. Consequently, the knockdown of each of the backbone proteins should prevent CRL function. In our experiments, the knockdown of the backbone Cul1 and Roc1, both essential for complex assembling, results in similar CM hypertrophy. However distinct F-box proteins could lead to either atrophic or hypertrophic effects in CM. Unexpectedly, the decrease in cell size after knockdown of Fbxo6 and Fbxl14 could not be confirmed by ^3^H-isoleucine incorporation, despite showing a stable nuclei count (Supplementary Figure S4) and no toxic effect assessed by MTT assays (Supplementary Figure S5). A possible explanation for these results could be yet unknown intracellular and metabolic changes upon depletion of these CRL subunits.

Besides Atrogin-1 (Fbxo32) and Fbxo25, published data about the role of the F-box proteins in cardiac diseases and growth control is still rare. So far only Fbxo9, being upregulated in a hindlimb unloading model (Wu et al., 2011) and the association of Fbxo30 in Nebulin-mediated myopathy are known (Sartori et al., 2013; Li et al., 2015). These observations are nevertheless mechanistically in accordance with our screening result. Interestingly Fbxw5 is involved several pathways such as MAP3K and JNK/p38 pathway (Minoda et al., 2009). Recently, Fbxw5 was shown to act as a negative regulator of pathological cardiac hypertrophy (Hui et al., 2021). Fbxw5 deficiency aggravated cardiac hypertrophy, while adeno-associated virus 9-mediated overexpression of Fbxw5 protected mice from hypertrophic stimuli, which is in line with our screening result for Fbxw5. The fact that other F-box proteins could regulate important cardiac pathways, such as Fbxo33 [regulation of growth factors by YB-1 (Lutz et al., 2006; Lyabin et al., 2014)], Fbxo45 [regulator of p73 and mTOR signalling (Peschiaroli et al., 2009; Saiga et al., 2009; Han et al., 2012; Chung et al., 2014)], Ddb1 [associated with hypertension phenotype in a specific population with a polymorphism that impairs the function of Gβ3 to target GRK2 ubiquitination (Zha et al., 2016)] opens exciting new insights that should be investigated in the future.

To date, this study identified other members of the UPS that bear the ability to modulate the hypertrophic response in mammal cardiomyocytes. However, it is tempting to speculate that F-box proteins play a more prominent role in heart hypertrophy and diseases; more studies are warranted to decipher their new potential role and intracellular mechanisms.

## Data Availability

The original contributions presented in the study are included in the article/Supplementary Material, further inquiries can be directed to the corresponding authors.
