# Supplementary material for: Identification of hypertrophy-modulating Cullin-RING ubiquitin ligases in primary cardiomyocytes
Source: Front Physiol. 2023 Mar 8;14:1134339. doi: 10.3389/fphys.2023.1134339 (PMC10030680; doi:10.3389/fphys.2023.1134339)
Supplement: Supplementary file 1 [file Table1.DOCX]

Supplementary Material

Identification of hypertrophy-modulating Cullin-RING ubiquitin ligases in primary cardiomyocytes

Maximillian Fischer ^1,2,3 *^, Moritz Jakab ^1,4^, Marc N. Hirt ^5,6^, Tessa R. Werner ^5,6^, Stefan Engelhardt ^1,3^, Antonio Sarikas ^7 *^

^1^Institute of Pharmacology and Toxicology, Technische Universität München, Biedersteiner Strasse 29, 80802 Munich, Germany.

^2^Present address: University Hospital Munich, Department of Cardiology, Medical Clinic and Polyclinic I, Marchioninistraße 15, 81377 Munich, Germany.

^3^DZHK (German Center for Cardiovascular Research), partner site Munich Heart Alliance, Munich, Germany.

^4^Present address: Division of Vascular Oncology and Metastasis, German Cancer Research Center Heidelberg, 69120 Heidelberg, Germany.

^5^Institute of Pharmacology, University Medical Center Hamburg-Eppendorf, Martinistr. 52, 20246 Hamburg, Germany.

^6^DZHK (German Center for Cardiovascular Research), partner site Hamburg/Kiel/Lübeck, Germany.

^7^Institute of Pharmacology and Toxicology, Paracelsus Medical University, Strubergasse 21, 5020, Salzburg, Austria.

*** Correspondence:**Dr. Maximilian Fischer
[maximilian.fischer@med.uni-muenchen.de](mailto:maximilian.fischer@med.uni-muenchen.de)
and
Prof. Antonio Sarikas
[antonio.sarikas@pmu.ac.at](mailto:antonio.sarikas@pmu.ac.at)

# Supplementary Figures and Tables

## Supplementary Figures


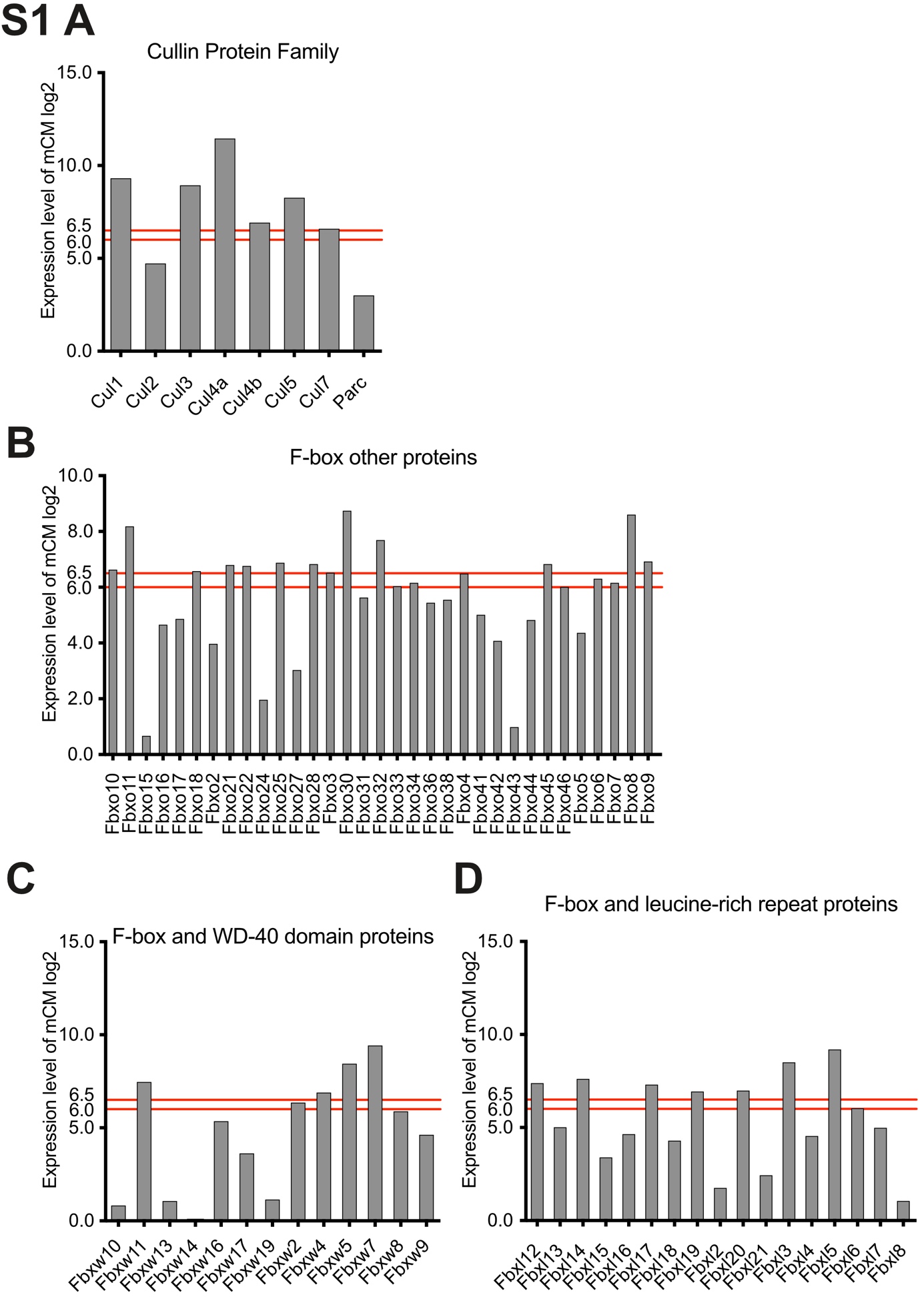


**Figure S1 description:**CRL subunit expression in microarray analysis. Analysis of the Netaffx microarray data in cardiomyocytes resulted in four target groups arranged by their protein family. **(A)** Expression level of Cullin protein family member, **(B)** F-box other proteins **(C),** F-box and WD-40 domain proteins, and **(D)** F-box and leucine-rich repeat proteins. Mean expression levels are displayed in log_2_ scale according to manufacturer protocol. Expression levels starting at 6.0 provide relevant protein synthesis and above at 6.5 argues for strong protein expression, according to the manufacturer.

**
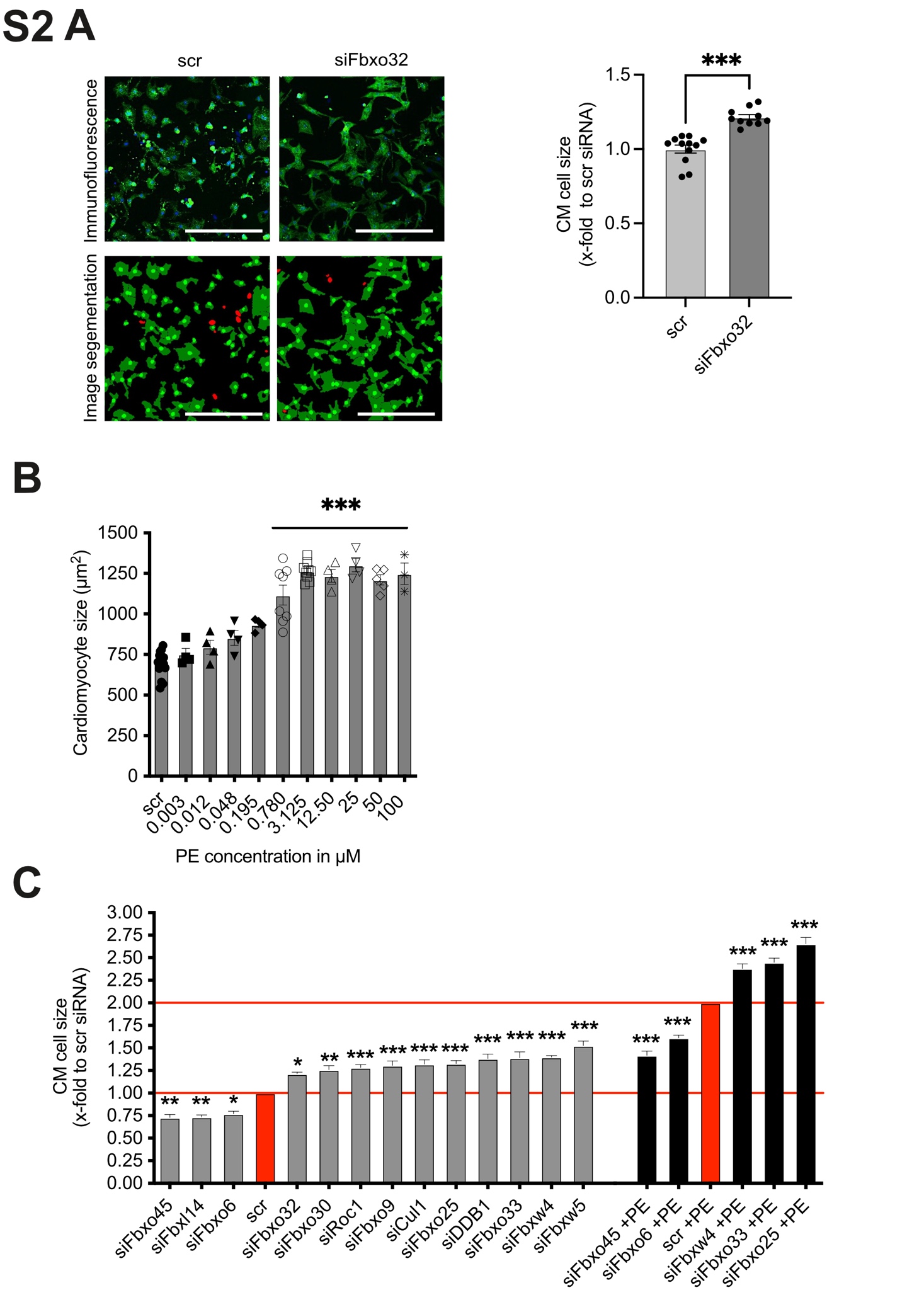
**

**Figure S2 description:**(**A**) Representative immunostaining and image segmentation of neonatal cardiomyocytes in cell culture after transfection of scramble siRNA or siRNA against *Fbxo32/Atrogin-1* mRNA; cardiomyocytes (α-actin in) were stained for the marker protein. Nuclei stained with DAPI in blue. Scale bar represents 250 μm. Histogram depicts quantitative data on the right panel. Effect of transfected siRNA on cardiomyocyte size under basal conditions. Each independent experiment with > 50,000 cells per group. Data are normalized to scrambled siRNA control. Independent experiments: N=3, n=10-12, unpaired Students t-test, ** P< 0.01, ***P < 0.001.

(**B**) Effect of increasing PE concentrations on induced CM hypertrophy. Cell size in µm^2^ was assessed by automated microscopy. N= 3 - 16 independent experiments. Statistically significant cell size change compared to scramble (scr) control is indicated. Data are mean ± SEM and statistically analyzed by one-way ANOVA with Bonferroni *post hoc test*, *** P<0.001.

(**C**) Illustrative overview of the identified screening targets sorted by increasing cell size effect. Basal conditions (left side) and PE stimulated condition (right side) are normalized to the corresponding scr or scr+PE control. Data are mean ± SEM and statistically analyzed by one-way ANOVA with Bonferroni *post hoc test.* Significance levels are reported to the corresponding baseline and PE-stimulated control * P<0.05, ** P< 0.01, ***P < 0.001.


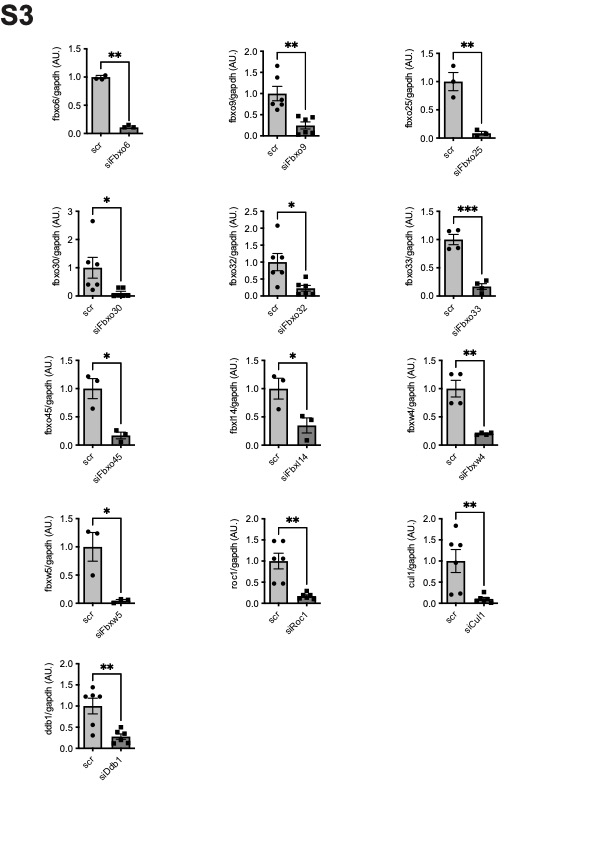
 **Figure S3 description:**Validation of each siRNA-mediated knockdown of mRNA by qPCR in cardiomyocytes after transfection. Scramble siRNA served as control. Data were normalized to *Gapdh* as housekeeping gene. Independent experiments: N=3, n=3-6. Data are mean ± SEM. Unpaired Student’s t-test, * P< 0.05, ** P< 0.01, *** P<0.001.

**Figure S4 description:**Nuclei count analysis after siRNA transfection. DAPI and α-actinin positive cells of the hypertrophy screening data were counted automatically and compared to scramble siRNA (scr) transfected cells. Each point represents the count of NRCMs in one picture taken by electronic microscopy in the hypertrophy assay. Independent experiments: N=3, n=19-48. Data are mean ± SEM. Unpaired Student’s t-test, ** P< 0.01, *** P<0.001.

**
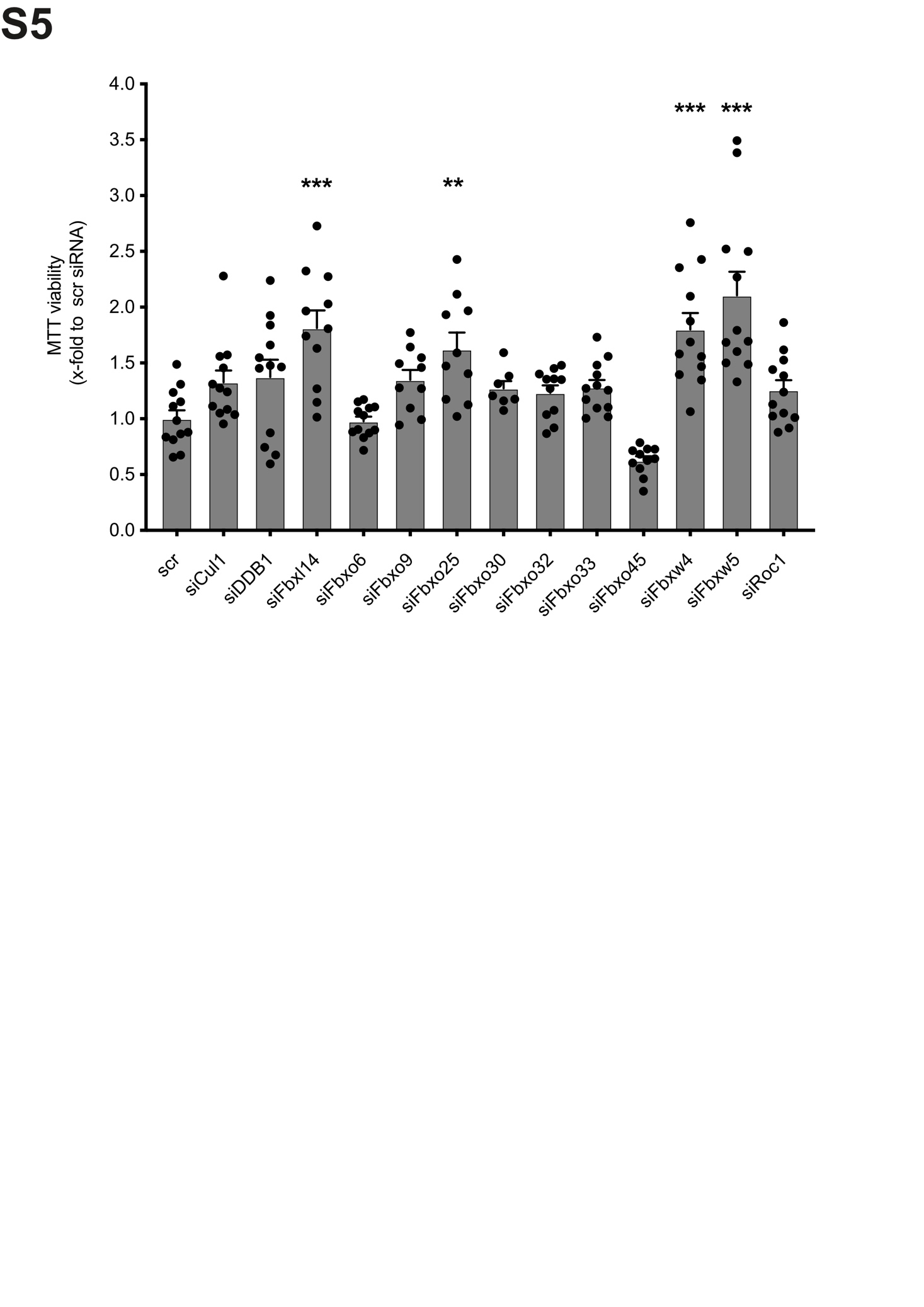
**

**Figure S5 description:**Cell viability after siRNA transfection was assessed by MTT assay. Uptake and metabolism of the MTT reaction were assessed to determine a toxic or beneficial effect on cardiomyocytes. Data were normalized to scramble siRNA control. Independent experiments: N=3, n=7-12. Data are mean ± SEM. and statistically analyzed by one-way ANOVA with Bonferroni *post hoc test*, ** P< 0.01, *** P<0.001.

## Supplementary Tables

**Table S1:**

| Group 1  (multiple measurements > 6.5 in CM)  Gene symbol (NCBI Gene ID) | Group 2  (multiple measurements > 6.0 – 6.49 in CM) | Group 3 |
| --- | --- | --- |
| Cul3 (301555)  Cul4b (302502)  Cul5 (64624)  Fbxl3 (306129)  Fbxl17 (316663)  Fbxl20 (64039)  Fbxo3 (690634)  Fbxo4 (310363)  Fbxo9 (300849)  Fbxo21 (360818)  Fbxo22 (300724)  Fbxo28 (305105)  Fbxo30 (308283)  Fbxo32 (171043)  Fbxo45 (ensemble ID RGD1311861)  Fbxw7 (100360914)  Fbxw11 (303024) | Cul1 (362356)  Cul4a (361181)  Cul7 (363191)  Fbxl5 (305424)  Fbxl12 (313782)  Fbxl14 (312675)  Fbxl19 (308999)  Fbxo6 (192351)  Fbxo8 (306436)  Fbxo10 (362511)  Fbxo11 (301674)  Fbxo18 (291293)  Fbxo25 (364637)  Fbxo33 (314157)  Fbxw2 (311881)  Fbxw4 (309444; like-protein 100911855)  Fbxw5 (362081)  Fbxw8 (304522) | Rbx1; Roc1 (300084)  Rnf7; Roc2 (300948)  Skp2 (294790)  DDB1 (64470)  Tcep2; Elongin B (81807)  Tcep1 ;Elongin C (64525)  BTRC; Fbxw1 (361765)  Cul9; PARC (316228) |

**Table S2:**

| Gene name | Fold-change mean cell size upon depletion (mean ± SEM)  without PE | Fold-change ^3^H-isoleucine incorporation upon depletion (mean ± SEM)  without PE |
| --- | --- | --- |
| Cul1 | 1.32 ± 0.05 | 1.13 ± 0.07 |
| Ddb1 | 1.38 ± 0.05 | 1.26 ± 0.05 |
| Fbxl14 | 0.74 ± 0.02 | 1.09 ± 0.07 |
| Fbxo6 | 0.77 ± 0.03 | 1.32 ± 0.12 |
| Fbxo9 | 1.31 ± 0.05 | 1.20 ± 0.05 |
| Fbxo25 | 1.37 ± 0.03 | 1.41 ± 0.12 |
| Fbxo30 | 1.26 ± 0.05 | 1.21 ± 0.05 |
| Fbxo32 | 1.21 ± 0.03 | 1.23 ± 0.06 |
| Fbxo33 | 1.39 ± 0.06 | 2.08 ± 0.29 |
| Fbxo45 | 0.73 ± 0.03 | 0.63 ± 0.03 |
| Fbxw4 | 1.39 ± 0.02 | 1.61 ± 0.15 |
| Fbxw5 | 1.53 ± 0.05 | 1.28 ± 0.03 |
| Roc1 | 1.29 ± 0.03 | 1.64 ± 0.13 |

| Gene name | Fold-change mean cell size upon depletion (mean ± SEM)  with PE | Fold-change ^3^H-isoleucine incorporation upon depletion (mean ± SEM)  with PE |
| --- | --- | --- |
| Fbxo6 | 0.81 ± 0.02 | 1.15 ± 0.06 |
| Fbxo25 | 1.32 ± 0.03 | 1.40 ± 0.05 |
| Fbxo33 | 1.32 ± 0.04 | 1.58 ± 0.06 |
| Fbxo45 | 0.71 ± 0.02 | 0.74 ± 0.07 |
| Fbxw4 | 1.19 ± 0.03 | 1.27 ± 0.08 |

**Table S3**

| Target gene (rat) |  | Sequence |
| --- | --- | --- |
| *Fbxo45* | forward | TGGCAGTGATAGGGATTGCT |
|  | reverse | TACCCACGCTCAAAGGCTAA |
| *Fbxo25* | forward | ACCAACCCTGTACATGCTCA |
|  | reverse | ACTGCTCTTTGGTCGGGTAA |
| *Fbxw4* | forward | AGGAACGAGTGAAGCTGTCT |
|  | reverse | CTTCTAGCTGCATCCAAGGC |
| *Fbxo6* | forward | CTTCGAGAGGGCTTTGTCAC |
|  | reverse | GCTCTCCACCTTCCACTCAT |
| *Fbxo33* | forward | GGTTTTATTGGCATGGCGGT |
|  | reverse | ATCCTGGTCGGCTAGTTCAC |
| *Roc1* | forward | CGGCGATGGATGTGGATAC |
|  | reverse | ACAATATCCCAGGCCCAGAG |
| *Fbxo9* | forward | TCTGCTGGGTCATTATCGCT |
|  | reverse | CAGCCCCACATGAAAGTTGT |
| *Fbxo30* | forward | TGAGGGACATTTGTGGCAGT |
|  | reverse | GCTTCCTCCCGCTTCTCTAT |
| *Fbxo32* | forward | TTGGATGAGAAAAGCGGCAC |
|  | reverse | CTTCTTGGCTGCAACATCGT |
| *Cul1* | forward | GGATGACCTCAGAGCTGGAA |
|  | reverse | CTCCAAGCCGACAAACTGAG |
| *Ddb1* | forward | GGTGCCATCATCATTGGACA |
|  | reverse | CCATCCATCTGCTCCTCCTT |
| *Fbxl14* | forward | GTCCCTCTCCCTTTGCTCTT |
|  | reverse | ATCCTAGTGCAGCCGTACAG |
| *Fbxw5* | forward | ATGGCTATATCTGGGACCGC |
|  | reverse | GCAAACCAGGAGAAGAAGGG |
| *Gapdh* | forward | TGACAACTCCCTCAAGATTGTCA |
|  | reverse | GGCATGGACTGTGGTCATGA |
| Rpl32 | forward | GCCAAGATCGTCAAAAAGA |
|  | reverse | GTCAATGCCTCTGGGTTT |
| Anp | forward | GGGCTTCTTCCTCTTCCT |
|  | reverse | TGAGACGGGTTGACTTCC |
| Bnp | forward | TGGGCAGAAGATAGACCG |
|  | reverse | GCAAGTTTGTGCTGGAAG |
| Target gene (mouse) |  | Sequence |
| *Fbxo25* | forward | GTGGGGAAGTCAGTGCTAGT |
|  | reverse | TGACAATGTCCCAGCCATCT |
| *Gapdh* | forward | GTGAAGGTCGGTGTGAACG |
|  | reverse | TCGTTGATGGCAACAATCTC |

**Table S4**

| ON-TARGETplus Rat siRNA- SMART pool | |
| --- | --- |
| Target gene | Sequence (5’ -> 3’) of oligonucleotides |
| *Cul3* | UUCAAGAAAUCCAGCGUAA  GAGAUCAAGUUGUACGGUA  UGACAGAAAACACGAGAUA  ACAGGAAGUAGAAACGAUA |
| *Cul4b* | UAACAGUAGUAACGAGAGA  CCAAAUUGAUGCUGCGAUC  UGAUUGAGAGAGAGCGGAA  AGACUUAGCCAAACGCCUA |
| *Cul5* | CUGCAAGAGAUAAGGCGUA  AAGGCAUGAUCAAGCGAAA  GGAAGACAGUAUUGUUCGA  UUAGAAACAAGACGAGAAU |
| *Fbxl3* | GCUUAGAACAUUUGCGCAU  ACAUGAUGCCUACGUGGUA  AUGCCUGACUUGUGGCGAU  AGACACACUUCCACACGAU |
| *Fbxl17* | UGAUAGCCAUUGGGCGAUA  GUUACAAGAUCUCGGACGA  GAAUAUACAUGCAGGAGAA  GUGUGGAGGUUAUCGCAAA |
| *Fbxl20* | CGGAUAGACCUGUUCGAUU  GUUCUCAAACAGUGACGAA  UCGUGGGUGUCUCGGAGUA  CAGAACUGCCCUCGGCUUA |
| *Fbxo3* | GCAGGAAACUAGCACGGAA  ACUCAGAUGUAGGAAGAUA  UGAAAGAGGGUGCGCGUGA  AGUUAGAGUUCUCACGCUA |
| *Fbxo4* | GAUACUUUCUGUUUCGAGA  UGUUCAGCCUACAGAGCGA  GCUCAUGAACUUCGUCUCA  AGAAUCUAAGCAUGCGAAA |
| *Fbxo9* | GUGAGAAGCUACACGGCGU  UGGCCGAUCUCCUGUCGUA  GGCACCAAGUGGAAUAUUA  AGGCGUAGGUUCCAGUAAU |
| *Fbxo21* | GAAUAUAAAGUUCGGCAGA  AGCACAAGAGGUACGGCUA  CCUGGAAGUACUACGCUAA  GGGCACGAGUGGAUCCGAA |
| *Fbxo22* | CGUGGUAGCUGAAGCGCUU  GGAGAGAGUGUGUGCGUAG  CAGAUGAAGGACAGCGUUA  GAAUUÜAACUCUCGAAAGA |
| *Fbxo28* | AAUCCAAACGACUGCGGAA  CCAGAUCAUAGGCGAGCAA  GCACAUUACAUGCGGAUUU  GCUUUAUGUCCUACGACGA |
| *Fbxo30* | CAGCCUAAGCAGCGUUCAA  AGACAAAGCAGUCGAUACU  AAGAAAAGGUGUGGCGAUU  UGAAAUUGUUGGAACGUGA |
| *Fbxo32* | GGCAGAUCCGCAAGCGAUU  ACAUGUGGGUGUAUCGAAU  CAUAAGACUCAUACGGGAA  ACUUUAAGCUUGUGCGAUG |
| *Fbxo45* | GAUAGGGAUUGCUACGAAA  GACUUUAGCCUUUGAGCGU  GGAGAGAGAAUCCGAGUCA  CAACAUGCCUUCAGCACGA |
| *Fbxw7* | GGUCAGCGGUCACGGGUAA  CGGGUGAAUUUAUCCGAAA  CGUUACAGUUUGACGGCAU  GCUCAGACGUGUCGAUACU |
| *Fbxw11* | GAUAAGUAAUGGAACGUCA  AGAUAAUACCAUCCGGUUA  AUUAAAGUGUGGAGCACGA  CAUCUUAUCUCACGAAUGU |
| *Cul1* | GAACAGUGCGAGUGACGAU  GAGCUCAGUUUGUCGGCUU  GCAAAGGGCCCUACGUUAA  AAGAAUAUUUGGAGCGAGU |
| *Cul4a* | CCAUGUGAGUAAACGUCUA  GACAGUGAGUUGCGAAGAA  CAAAAUGGCCACCGGCAUA  GCUGAUUGGGCGUGAACGA |
| *Cul7* | CUACAUGGUUCGUGCACAA  CCGCAUGCUAGCCGAGUAA  UCAAGAAGUUAAACGCAGA  AGAUGAGGCUGGCGCUGAA |
| *Fbxl5* | GGGACGAAGAUGCCGAUAU  CCAAGAGACUGGACGAGUA  GCUGAAGAUUUGGCCGAUA  CAAAAUGAGUCUUCGGCAU |
| *Fbxl12* | CUGCCUAGUUAUCGUCAGA  GCUGGAAUGCAUCGUGCUA  GCUGCGAAAUCUCGAUGAU  CCGAGAUGUGCGCAAGAUU |
| *Fbxl14* | UGGCCAUGAUCUUCGGCUA  GCAUAGACCUGUACGGCUG  GUAGAAAGCMGCCCGACU  CGACMCAUCAGCGACACU |
| *Fbxl19* | GGGAGMGCUGGAGCGUUU  GCAUGMGCAGUCGUGCCU  UGCCAUUUCUGCCGAGACA  GCUGAGGGUGUUAUCMUU |
| *Fbxo6* | CCUGAGAUCAUGGCGGAUA  GGCCGGACAUUGUGGUUAA  UCUUAUGGGAUGUGCCUCA  GAGGAAGAUACUCAGCUUU |
| *Fbxo8* | CCAAAUGCAUUGCGAGAAU  AAGCACAAUUGUUCGAACU  GAGAGAGUUUAUUCGAAAU  UCACACCAGUCACCGGAAA |
| *Fbxo10* | GGACAGUACCCGUUGGCGA  CAAGGAAGCCAUGGCGCUA  AGGAAGAAGGAGCGACGUA  GAAUUUGUGGGCAGCGAAA |
| *Fbxo11* | UCCAAUUAUUAGACGGAAU  AGAAAAGGCUGUUAGUAGA  ACUAAGAAGAAAUCGGAUA  AGGUAAAAGCCUACGCUAA |
| *Fbxo18* | AGAAUCGCCUGCACGGAUA  GGUUAGAGUUUGACACGGU  GGUACUUGAUCAACGAAGA  CAGCAAAGUGGACGGCAUU |
| *Fbxo25* | GCUGAACGCCACACGUAGA  GACUUGAGCUCGACCCUUU  GGGAAAUCAGUGUUAGUUG  UGGAUAAAAUCGUGCAAAA |
| *Fbxo33* | CAGUCGAACUAGAGCGCUU  CAAAGAACGUAGUCGGGUA  UCGAGUUUCUCAUGCGCAA  GCGAGAGUCCUGACCGACA |
| *Fbxw2* | GUGCAGACAAGUACGAGAU  AGGUUUACUUGAAGGCUAU  UCUCUAAGCAGUGGAAUAA  GGCAGUGUGUUUACGGCAU |
| *Fbxw4* | CCAGUAAAGGAACGAGUGA  CUUACAACCUCCACGUCUU  CACAAGAGCAGGAGCGUCA  GAAGAAGGGCCGAGACGCU |
| *Fbxw5* | UUAUAGCAGUAACGCCUUA  GGGAGCAGUUCUACCGCUA  CAAGGAAGGUUUACGGCGU  CCUUUCAUACUCAGCCAAA |
| *Fbxw8* | GCAAAGGUGCCGUGAGCGA  CUUAUGAACUGGCGAUCAA  CAUGGAAGGUGAUUGCCGA  ACGAGAAGGUGCUGCGAAA |
| *Roc1* | CCCAUUUGUUACUCGUUUA  GGAUCUUUGUAUCGAGUGU  AAUCUUGGCUGGCAAUAGA  GGUACUUGGGAAUUAGGUA |
| *Roc2* | GCUGGGACGUUGAGUGCGA  CAAGAAGUGGAACGCGGUA  GGGUUAACUUGUACUGAGU  GGAAGGUUGUAUUGUAUGU |
| *Skp2* | GGCCUAAGCUCAAUCGAGA  GGUAUCAAAUGCCGACUGA  UGUCAAACCUCCACGGGAU  CAGCCGGUGCUACGAUAUA |
| *Ddb1* | CCACUGAUGAGGAGCGGCA  ACACUUUACUUCAGCGGAA  CCUCAGGAGCUCCGGCAAA  AGAUAUGGAAGGACGGCUU |
| *Elongin-B* | CCUUUGAAGCGCUGCGUAU  GCACGGUGUUCGAACUGAA  UGAACAAGCUGUGCAGUGA  CCACAGGAUUCUGGAGGCA |
| *Elongin-C* | CAGGUCAGUUUGCGGAGAA  GCCAUGUAUGUGAAAUUAA  GCAUUAACAUCAGGAACAA  UCGAAAGUGUGCAUGUAUU |
| *BTRC/Fbxw1* | CCAGAUAAAUAACGCGACA  CGGUCAAUGUUGUCGACUU  GGACAUAGAGUGCGGUGCA  UCAGAGUGUGGGACGUAAA |
| *Cul9* | ACAUGAGGGCAUCGAGCAA  GAACAGAGUUUUACGCCAU  GCACAAAGACUAUGCGGUA  GGACAGGAAGCCAGCGCAU |
