# Supplementary material for: Identification of hypertrophy-modulating Cullin-RING ubiquitin ligases in primary cardiomyocytes
Source: Front Physiol. 2023 Mar 8;14:1134339. doi: 10.3389/fphys.2023.1134339 (PMC10030680; doi:10.3389/fphys.2023.1134339)
Supplement: Supplementary file 2 [file DataSheet1.PDF]

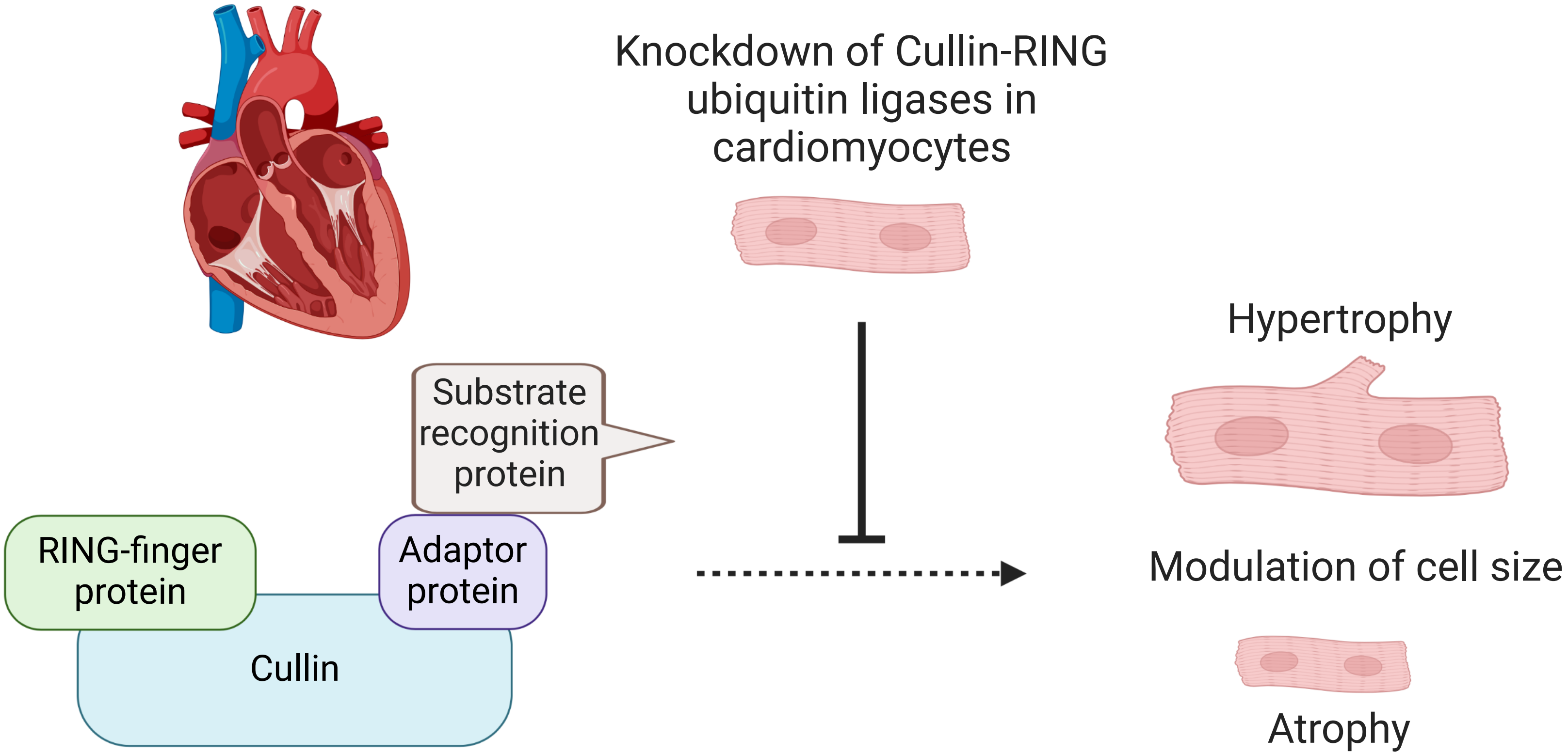
Knockdown of Cullin-RING ubiquitin ligases in cardiomyocytes
Substrate recognition protein
RING-finger protein
Adaptor protein
Cullin
Hypertrophy
Modulation of cell size
Atrophy
